# Brain imaging and neuropsychological assessment of individuals recovered from a mild to moderate SARS-CoV-2 infection

**DOI:** 10.1073/pnas.2217232120

**Published:** 2023-05-23

**Authors:** Marvin Petersen, Felix Leonard Nägele, Carola Mayer, Maximilian Schell, Elina Petersen, Simone Kühn, Jürgen Gallinat, Jens Fiehler, Ofer Pasternak, Jakob Matschke, Markus Glatzel, Raphael Twerenbold, Christian Gerloff, Götz Thomalla, Bastian Cheng

**Affiliations:** ^a^Department of Neurology, University Medical Center Hamburg-Eppendorf, 20251 Hamburg, Germany; ^b^Department of Cardiology, University Heart and Vascular Center, 20251 Hamburg, Germany; ^c^Population Health Research Department, University Heart and Vascular Center, 20251 Hamburg, Germany; ^d^Department of Psychiatry and Psychotherapy, University Medical Center Hamburg-Eppendorf, 20251 Hamburg, Germany; ^e^Department of Neuroradiology, University Medical Center Hamburg-Eppendorf, 20251 Hamburg, Germany; ^f^Department of Psychiatry, Brigham and Women’s Hospital, Harvard Medical School, 202115 Boston, MA; ^g^Department of Radiology, Brigham and Women’s Hospital, Harvard Medical School, 202 Boston, MA; ^h^Institute of Neuropathology, University Center Hamburg-Eppendorf, Hamburg, 20251 Gemany; ^i^German Center for Cardiovascular Research, Partner site Hamburg/Kiel/Luebeck, 20251 Hamburg, Germany; ^j^University Center of Cardiovascular Science, University Heart and Vascular Center, 202115 Hamburg, Germany

**Keywords:** COVID-19, neuroimaging, diffusion MRI, structural MRI, neuropsychological assessment

## Abstract

As severe acute respiratory syndrome coronavirus type 2 (SARS-CoV-2) infections have been shown to affect the central nervous system, the investigation of associated alterations of brain structure and neuropsychological sequelae is crucial to help address future health care needs. Therefore, we performed a comprehensive neuroimaging and neuropsychological assessment of 223 nonvaccinated individuals recovered from a mild to moderate SARS-CoV-2 infection (100 female/123 male, age [years], mean ± SD, 55.54 ± 7.07; median 9.7 mo after infection) in comparison with 223 matched controls (93 female/130 male, 55.74 ± 6.60) within the framework of the Hamburg City Health Study. Primary study outcomes were advanced diffusion MRI measures of white matter microstructure, cortical thickness, white matter hyperintensity load, and neuropsychological test scores. Among all 11 MRI markers tested, significant differences were found in global measures of mean diffusivity (MD) and extracellular free water which were elevated in the white matter of post-SARS-CoV-2 individuals compared to matched controls (free water: 0.148 ± 0.018 vs. 0.142 ± 0.017, *P *< 0.001; MD [10^−3^ mm^2^/s]: 0.747 ± 0.021 vs. 0.740 ± 0.020, *P *< 0.001). Group classification accuracy based on diffusion imaging markers was up to 80%. Neuropsychological test scores did not significantly differ between groups. Collectively, our findings suggest that subtle changes in white matter extracellular water content last beyond the acute infection with SARS-CoV-2. However, in our sample, a mild to moderate SARS-CoV-2 infection was not associated with neuropsychological deficits, significant changes in cortical structure, or vascular lesions several months after recovery. External validation of our findings and longitudinal follow-up investigations are needed.

## Significance

In this case–control study, we demonstrate that non-vaccinated individuals recovered from a mild to moderate severe acute respiratory syndrome coronavirus type 2 (SARS-CoV-2) infection show significant alterations of the cerebral white matter identified by diffusion-weighted imaging, such as global increases in extracellular free water and mean diffusivity. Despite the observed brain white matter alterations in this sample, a mild to moderate SARS-CoV-2 infection was not associated with worse cognitive functions within the first year after recovery. Collectively, our findings indicate the presence of a prolonged neuroinflammatory response to the initial viral infection. Further longitudinal research is necessary to elucidate the link between brain alterations and clinical features of post-SARS-CoV-2 individuals.

As the number of patients recovering from an acute infection with the severe acute respiratory syndrome coronavirus type 2 (SARS-CoV-2) grows, the study of its long-term consequences on health outcomes has gained much attention (1–4).

It is widely recognized that coronavirus disease 2019 (COVID-19) caused by SARS-CoV-2 not only leads to respiratory dysfunction but also impacts various other organ systems during the acute phase and well beyond (1, 5, 6). Neurological symptoms, such as headache, fatigue, memory, and attention deficits, may significantly impede well-being in individuals suffering from COVID-19 sequelae (4, 7, 8). Advancing our understanding of the underlying pathological mechanisms will be crucial for addressing future health care needs.

Different potential mechanisms have been suggested to be involved in the development and persistence of neurological symptoms in patients with COVID-19. Postmortem histopathological and molecular studies have demonstrated viral neurotropism, signs of neuroinflammation (9, 10), neurodegeneration (11), demyelination (12), axonal disruption (13), and micro- and macrovascular damage (14, 15). However, most studies were conducted in patients with severe COVID-19, whereas histopathological findings from individuals with mild to moderate courses are lacking.

In vivo studies applying modern brain imaging joined by comprehensive clinical and neuropsychological assessment are scarce. Recent preliminary evidence from the UK Biobank suggests cortical thickness reductions in the olfactory and limbic network, as well as neurocognitive decline in former COVID-19 patients, although these findings still need to be replicated in independent datasets (11). The majority of remaining studies focused on visually apparent pathological findings such as intracranial hemorrhage, stroke, or white matter hyperintensities in small case series or single case reports of more severely affected patients (16–19). Taken together, current evidence is of limited transferability to patients with a mild to moderate SARS-CoV-2 infection, therefore necessitating further investigations.

In order to address this research need, we studied 223 nonvaccinated individuals in median 289 d after recovery from mainly mild to moderate SARS-CoV-2 infections in a retrospective, cross-sectional case–control design. We leveraged advanced MRI techniques enabling the study of imaging phenotypes associated with neurodegeneration, atrophy, myelin/cellular disruption, inflammation, and vascular damage (20–23). Moreover, study participants received a comprehensive clinical and neuropsychological assessment. Building upon our previous multiorgan assessment in this cohort (1), here, we provide a detailed in vivo assessment of the cerebral white and gray matter, as well as neuropsychological outcomes in former COVID-19 patients.

## Results

### Sample Characteristics.

We examined participants of the Hamburg City Health Study (HCHS) and its COVID Program. Imaging data were available for 230 post-SARS-CoV-2 individuals. Following quality assessment (QA), in total, 7 post-SARS-CoV-2 individuals had to be excluded, leaving 223 cases for propensity score matching with healthy controls who had passed QA. The results of the matching procedure are visualized in *SI Appendix*, Fig. S1. The final sample included 223 matched controls (93 female, age in years, mean ± SD, 55.74 ± 6.60) and 223 post-SARS-CoV-2 individuals (100 female, 55.54 ± 7.07; see Table 1). Of the latter, the majority had a mild to moderate course of COVID-19 (without symptoms, n = 7; mild symptoms, n = 125; moderate symptoms, n = 67), 18 were hospitalized, and none required mechanical ventilation or intensive care unit treatment. There were no significant differences between post-SARS-CoV-2 individuals and matched controls regarding age, sex, years of education, and cardiovascular risk factors.

**Table 1.** Baseline characteristics of post-SARS-CoV-2 individuals and matched controls

	Post-SARS-CoV-2 individuals (N = 223)	Matched controls (N = 223)	*P_uncorr_*
Demographics			
Age in years, mean ± SD	55.54 ± 7.07	55.74 ± 6.60	0.76
Female sex, N (%)	100 (44.8)	93 (41.7)	0.56
Education in years, mean ± SD	15.70 ± 2.56	15.67 ± 2.86	0.91
COVID-19-specific characteristics			
Self-reported disease severity at the time of infection			
Asymptomatic, N (%)	7 (3.1)		
Mild, N (%)	125 (56.1)		
Moderate, nonhospitalized, N (%)	67 (30.0)		
Moderate, hospital ized, without ICT, N (%)	18 (8.1)		
Days between the first positive SARS-CoV-2 PCR test and study enrollment, median (IQR)	289 (163, 318)		
Cardiovascular risk factors			
Hypertension^*^, N (%)	131 (58.7)	121 (54.3)	0.39
Dyslipidemia^†^, N (%)	54 (24.2)	51 (22.9)	0.82
Diabetes mellitus^‡^, N (%)	16 (7.2)	13 (5.8)	0.70
Smoking, ever, N (%)	107 (48.0)	105 (47.1)	0.92

*Abbreviations*: COVID-19 = coronavirus disease 2019, ICT = intensive care treatment, IQR = interquartile range, PCR = polymerase chain reaction, post-SARS-CoV-2 individuals = individuals who recovered from a severe acute respiratory coronavirus type 2 infection, SD = standard deviation.

^*^Prevalence of hypertension was defined as blood pressure ≥140/90 mmHg, intake of antihypertensive medication, or self-report.

^†^Prevalence of dyslipidemia was defined as LDL cholesterol/HDL cholesterol ratio >3.5 or intake of lipid-lowering therapies.

^‡^Prevalence of diabetes mellitus was defined as fasting blood glucose level >126 mg/dL or self-report.

### Clinical Data.

Although post-SARS-CoV-2 individuals showed nominally better test performances in Verbal Fluency (VF), Mini-Mental State Examination (MMSE), and clock drawing test (CDT) compared to matched controls, after Bonferroni correction for multiple comparisons, no significant group differences remained in any neuropsychological test score, including those associated with executive functioning, memory, psychosocial, and neurological symptom burden (Table 2).

**Table 2.** Results of clinical and neuropsychological assessments of post-SARS-CoV-2 individuals compared to matched controls

Clinical measure^*^	Post-SARS-CoV-2 individuals	Matched controls	*P_uncorr_* ^†^	*P_bonf_* ^‡^	*F*
Neurocognition					
TMT-A in seconds	31.89 ± 10.60 (212)	33.71 ± 11.67 (190)	0.12	>0.99	2.40
TMT-B in seconds	68.50 ± 22.69 (212)	70.89 ± 25.57 (187)	0.37	>0.99	0.81
VF	28.03 ± 6.04 (212)	26.43 ± 7.15 (212)	0.02	0.14	5.94
WLR	8.52 ± 1.63 (210)	8.32 ± 1.61 (204)	0.25	>0.99	1.33
MMSE	28.37 ± 1.26 (211)	28.02 ± 1.72 (210)	0.02	0.19	5.34
CDT	6.75 ± 0.78 (212)	6.57 ± 1.03 (214)	0.04	0.37	4.20
Psychosocial symptom burden					
PHQ-9	3.94 ± 3.74 (212)	3.91 ± 3.77 (215)	0.97	>0.99	<0.01
GAD-7	2.94 ± 3.28 (212)	2.80 ± 3.06 (215)	0.67	>0.99	0.18
Neurological symptom burden					
PHQ-15^§^	2.13 ± 1.83 (212)	1.83 ± 1.73 (215)	0.09	0.82	2.86

*Abbreviations*: CDT = clock drawing test, GAD = general anxiety disorder, MMSE = Mini-Mental State Examination, PHQ = Patient Health Questionnaire, post-SARS-CoV-2 individuals = individuals who recovered from a severe acute respiratory coronavirus type 2 infection, SD = standard deviation, TMT-A = Trail Making Test Part A, TMT-B = TMT Part B, VF = verbal fluency, WLR = word list recall.

^*^Presented as mean ± SD (N).

^†^Uncorrected *P* values of analyses of covariance, adjusted for age, sex, and years of education.

^‡^Bonferroni-corrected *P* values of analyses of covariance, adjusted for age, sex, and years of education (considering 9 comparisons).

^§^PHQ-15 items: headache, dizziness, fatigue, and sleep disturbances.

### Imaging.

**Fig. 1.**
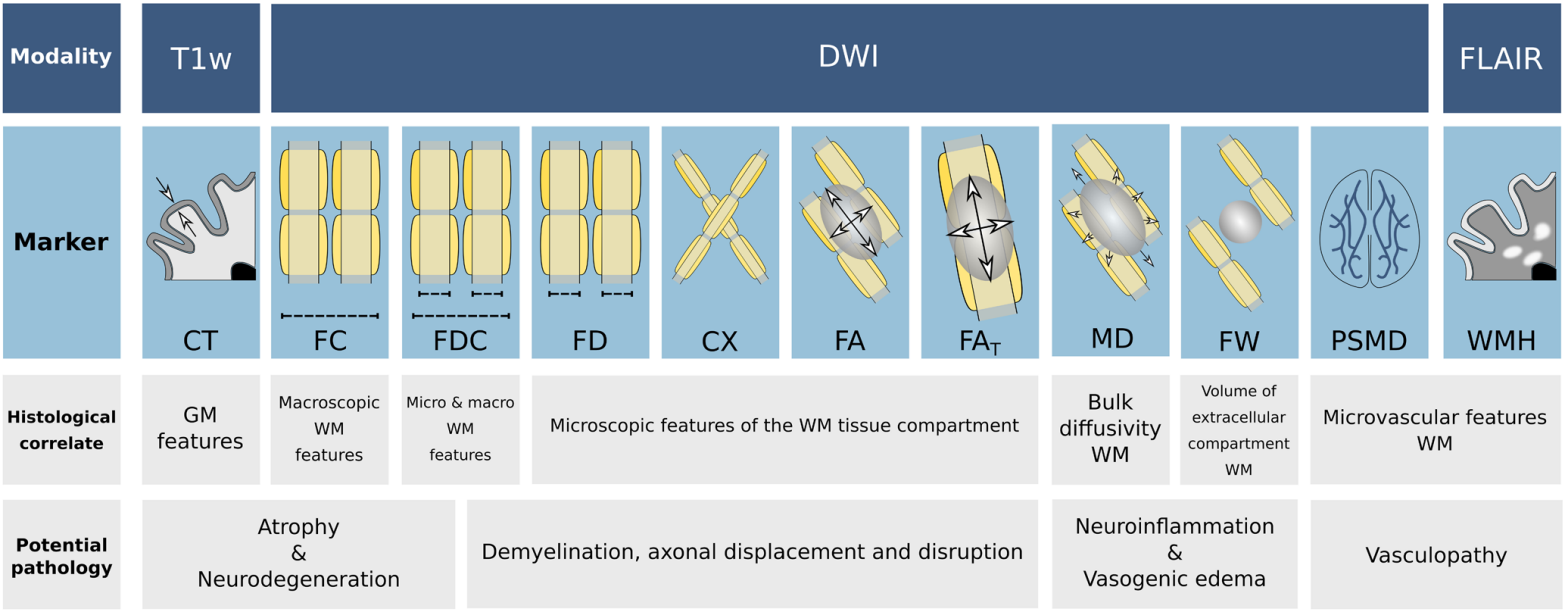
Schematic illustration of the investigated imaging markers. To assess the cerebral gray and white matter, micro- and macrostructural imaging markers were derived. The first row of the schematic describes the imaging sequences utilized to derive the imaging markers below. The second row presents diagrammatic illustrations of the markers: CT was determined as the distance between the pial surface and white matter/gray matter boundary; FC represents the macroscopic white matter fiber-bundle diameter; FD reflects the microscopic intraaxonal volume; as the combinatorial measure of FC and FD, FDC simultaneously assesses micro- and macroscopic alterations of white matter tracts; CX measures the intricacy of fiber configurations within a voxel; FA measures the directional preference of diffusion; MD denotes the molecular diffusion rate; free-water imaging enables the adjustment of traditional diffusion tensor imaging markers for extracellular diffusion signal, which increases their tissue specificity (FA_T_); FW represents the volume of the extracellular compartment; PSMD was calculated as the difference of the 95th and 5th percentile of skeletonized MD values; WMH load represents the white matter hyperintensity volume normalized by the total intracranial volume. Histological interpretations of the respective imaging markers and their potential sensitivity for pathologies are described in the third and fourth row, respectively. *Abbreviations*: CT = cortical thickness, CX = complexity, FA = fractional anisotropy, FA_T_ = FA of the tissue, FD = fiber density, FDC = fiber density and cross-section, FLAIR = fluid-attenuated inversion recovery, FW = free water, Log. FC = logarithm of fiber cross-section, MD = mean diffusivity, PSMD = peak width of skeletonized MD, WMH = white matter hyperintensity.

**Fig. 2.**
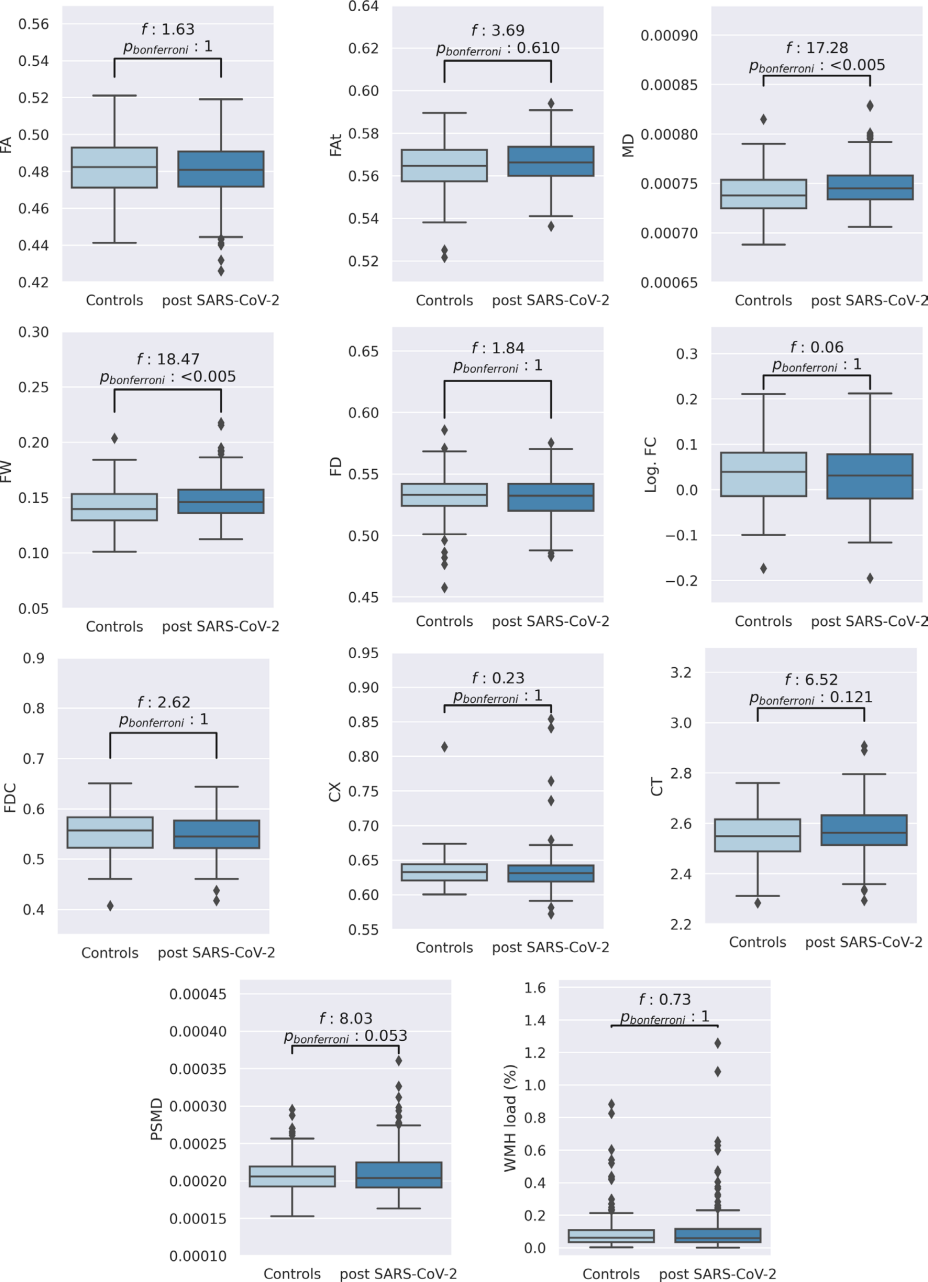
Group comparison of neuroimaging indices on a global scale. Boxplots of averaged imaging measures and the corresponding statistical results (F-statistics and Bonferroni-corrected *P* values for 11 comparisons) from the ANCOVAs comparing matched controls with post-SARS-CoV-2 individuals adjusted for age, sex, and years of education. *Abbreviations*: CT = cortical thickness, CX = complexity, FA = fractional anisotropy, FA_T_ = FA of the tissue, FD = fiber density, FDC = fiber density and cross-section, FW = free water, Log. FC = logarithm of fiber cross-section, MD = mean diffusivity, post-SARS-CoV-2 = individuals who recovered from a severe acute respiratory coronavirus type 2 infection, PSMD = peak width of skeletonized MD, WMH = white matter hyperintensity.

We first conducted analyses of covariance, adjusted for sex, age, and education, to test for group differences in imaging markers averaged across the entire white matter or cortical gray matter in the case of cortical thickness. A schematic illustration of the imaging markers under investigation is shown in Fig. 1. Post-SARS-CoV-2 individuals exhibited higher global extracellular free water and mean diffusivity (MD) in the cerebral white matter relative to matched controls, markers associated with immune activation and atrophy (mean ± SD, free water: 0.148 ± 0.018 vs. 0.142 ± 0.017, *F *= 18.47, *P_bonf_* < 0.001; MD [10^−3^ mm^2^/s]: 0.747 ± 0.021 vs. 0.740 ± 0.020, *F *= 17.28, *P_bonf_* < 0.001) (Fig. 2 and *SI Appendix*, Table S1). To aid the biological interpretation, we converted the mean group differences in free water and MD to units of “years of healthy aging” using the estimates of linear regressions with age in the matched control group (free water: *beta* = 0.0009, *P *< 0.001, MD: *beta* = 0.000001, *P *< 0.001). Considering the mean differences between groups of 0.006 in free water and 0.000007 in MD, this resulted in group differences of 6.67 and 7 “years of healthy aging”, respectively. While peak width of skeletonized mean diffusivity (PSMD) (*P_uncorr _*= 0.005), a marker of cerebral small vessel disease, and cortical thickness (*P_uncorr _*= 0.01) were nominally increased in post-SARS-CoV-2 individuals, both measures, as well as the remaining averaged imaging markers of white matter fiber structure, were not significantly different between groups after Bonferroni correction (Fig. 2 and *SI Appendix*, Table S1).

**Fig. 3.**
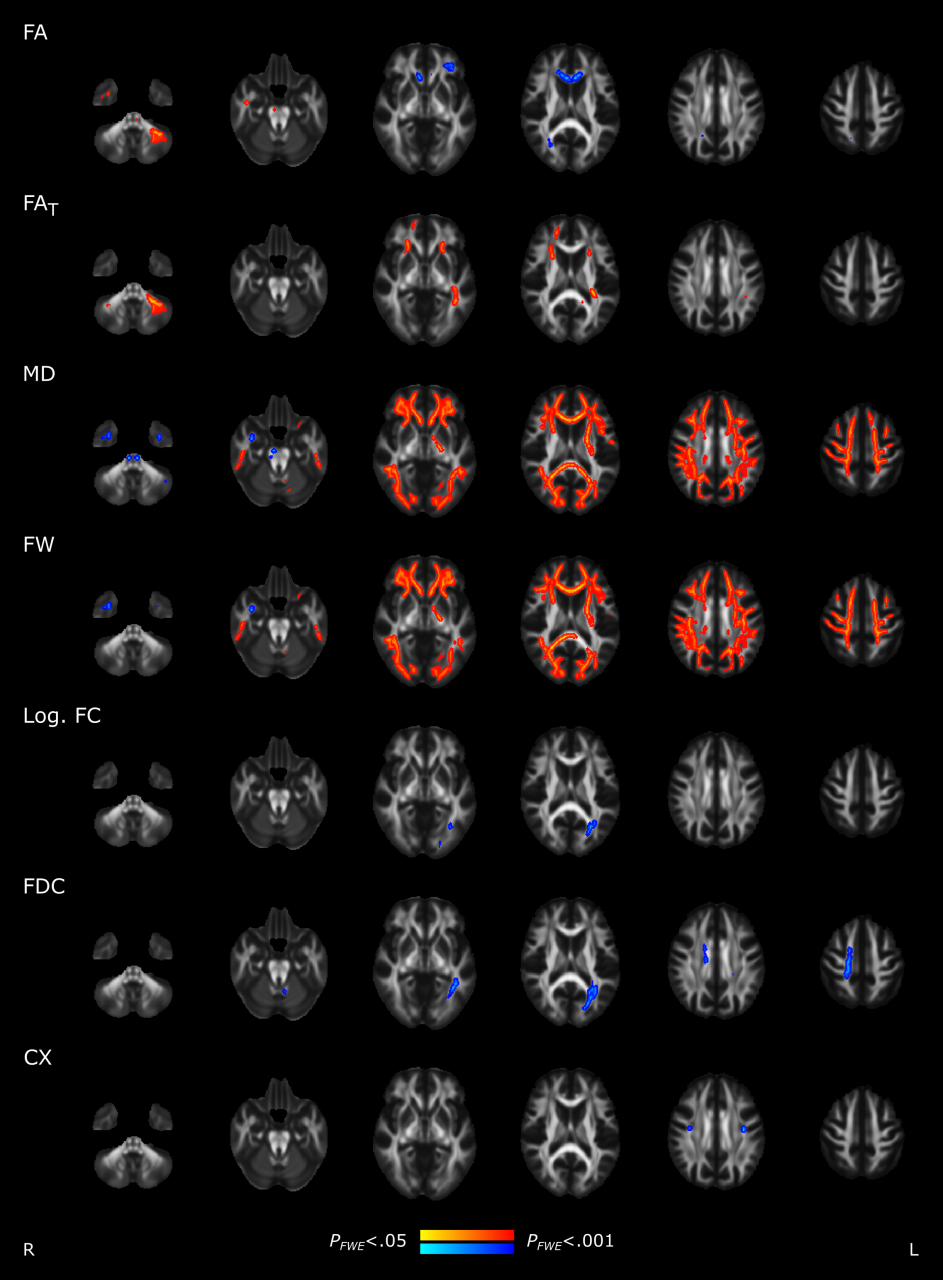
Group comparison of skeletonized diffusion indices. Skeleton voxels that significantly differed between groups are highlighted by colors: post-SARS-CoV-2 individuals > matched controls, red; post-SARS-CoV-2 individuals < matched controls, blue. *Abbreviations*: CX = complexity, FA = fractional anisotropy, FA_T_ = FA of the tissue, FD = fiber density, FDC = fiber density and cross-section, FW = free water, FWE = family-wise error corrected, Log. FC = logarithm of fiber cross-section, MD = mean diffusivity, post-SARS-CoV-2 = individuals who recovered from a severe acute respiratory coronavirus type 2 infection.

To detect spatial patterns of brain structural alterations, we additionally performed vertex- and voxel-wise analyses of gray and white matter imaging markers. Vertex-wise comparisons of cortical thickness did not reveal significant differences between matched controls and post-SARS-CoV-2 participants. Voxel-wise statistics on the entire white matter skeleton, a representation of major white matter fiber bundles, revealed predominant free water and MD increases in the white matter skeleton of post-SARS-CoV-2 subjects encompassing all brain lobes, compared to very localized changes in other diffusion markers (Fig. 3 and *SI Appendix*, Table S2). More specifically, the conventional diffusion tensor imaging (DTI) markers fractional anisotropy (FA) and MD showed significant differences between groups, with FA increases in 0.8% and decreases in 1.2% of the skeleton in cases relative to healthy controls. MD was significantly increased in 41.3% and decreased in 0.1% of the skeleton of post-SARS-CoV-2 participants. Employing free-water imaging, post-SARS-CoV-2 individuals showed significant free-water elevations in 38.3% and reductions in 0.4% of the skeleton, as well as FA of the tissue (FA_T_) elevations in 3.3% of the skeleton, but no FA_T_ reductions. Alterations of the remaining diffusion markers were of even less spatial extent (<3%).

**Fig. 4.**
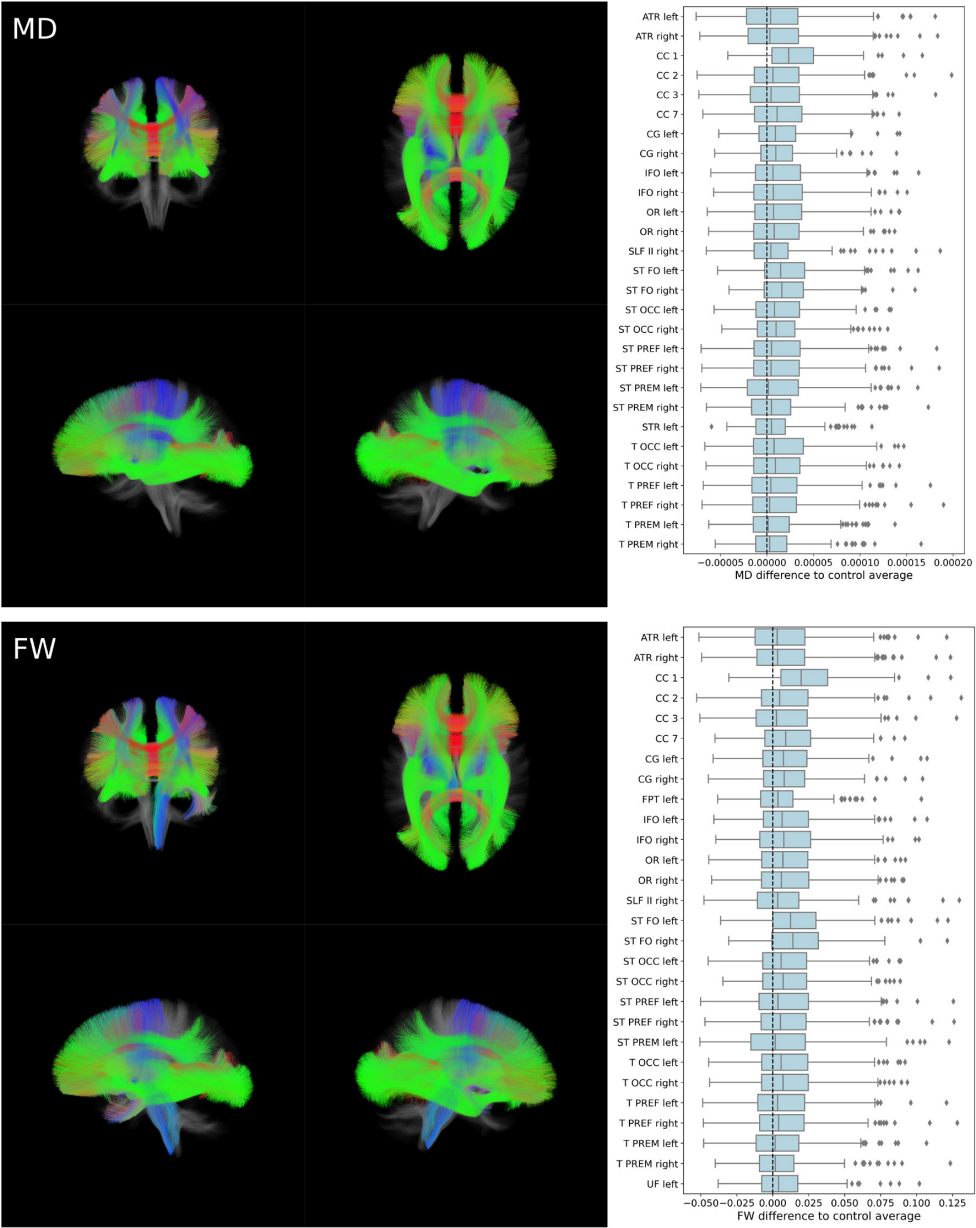
Tract of interest analysis of mean diffusivity and free water. *Left*: 3D visualization of investigated white matter fiber tracts represented as streamline bundles. Tracts that significantly differed between groups are highlighted by colors encoding directionality. Presented perspectives are coronal, anterior to posterior (*Upper* *Left*); axial, superior to inferior (*Upper* *Right*); sagittal, left to right (*Lower* *Left*); sagittal, right to left (*Lower* *Right*). *Right*: Boxplots displaying differences of post-SARS-CoV-2 individuals to the control group average. Only data of tracts that significantly differed after Bonferroni correction (71 comparisons) are shown. Boxplots considering all tracts reconstructed by TractSeg can be found in *SI Appendix*. *Abbreviations*: ATR = anterior thalamic radiation, CC = corpus callosum, CG = cingulum, FPT = frontopontine tract, FW = free water, IFO = inferior fronto-occipital fascicle, MD = mean diffusivity, OR = optic radiation, post-SARS-CoV-2 = individuals who recovered from a severe acute respiratory coronavirus type 2 infection, SLF = superior longitudinal fascicle, ST FO = striato-fronto-orbital tract, ST OCC = striato-occipital tract, ST PREF = striato-prefrontal tract, ST PREM = striato-premotor tract, STR = superior thalamic radiation, T OCC = thalamo-occipital tract, T PREF = thalamo-prefrontal tract, T PREM = thalamo-premotor tract, UF = uncinate fascicle.

We complemented voxel-based approaches by an assessment of diffusion indices averaged within anatomically predefined white matter fiber tracts in a tract of interest analysis. The tract-of-interest approach revealed widespread effects of significantly higher MD and FW in multiple association, commissural, and projection tracts including the anterior thalamic radiation, the corpus callosum, cingular projections, the frontopontine tract, the inferior fronto-occipital fascicle, the optic radiation, the superior longitudinal fascicle, the striatal as well as thalamic projections, and the uncinate fascicle (Fig. 4). FA and FA_T_ also significantly differed in association and commissural tracts. Corresponding details to FA and FA_T_ results as well as boxplots displaying data of all tracts investigated are provided in *SI Appendix*.

### Associations between Clinical and Imaging Data.

Exploratory regression analyses were performed between clinical measures and averaged imaging markers that showed significant group differences, i.e., free water and MD.

Linear regression revealed a significant positive association of free water with Trail Making Test Part A (TMT-A) (*P *= 0.008) and Part B (TMT-B) (*P *< 0.001), as well as significant negative associations of free water with VF (*P *= 0.003) and Word List Recall (WLR) (*P *< 0.001) in the entire sample (*SI Appendix*, Table S3). MMSE, CDT, Patient Health Questionnaire-9 (PHQ-9), General Anxiety Disorder-7 (GAD-7), and PHQ-15 scores were not significantly correlated with free water. Moreover, we observed significant group × free-water interactions for VF (*P *= 0.006), WLR (*P *= 0.02), MMSE (*P *= 0.02), and CDT (*P *= 0.04). Post hoc Spearman correlations performed for matched controls and post-SARS-CoV-2 individuals, separately, confirmed positive associations with TMT-A (*rho *= 0.20, *P *= 0.004) and TMT-B (*rho *= 0.22, *P *= 0.001), as well as negative correlations with VF (*rho *= −0.23, *P *< 0.001) and WLR (*rho *= −0.25, *P *< 0.001) in the post-SARS-CoV-2 group. However, among all neuropsychological measures, free water was only significantly correlated with TMT-B (*rho *= 0.15, *P *= 0.04) in the group of matched controls (*SI Appendix*, Table S3 and Fig. S5).

Results for MD were very similar. Linear regression analyses showed significant positive associations of MD with TMT-A (*P* = 0.03) and TMT-B (*P *= 0.001), as well as negative associations of MD with VF (*P *= 0.01) and WLR (*P *< 0.001) in the combined group of matched controls and post-SARS-CoV-2 individuals. Further, significant group × MD interactions were present for TMT-A, VF, WLR, MMSE, and CDT (*SI Appendix*, Table S4). Post hoc Spearman correlations revealed significant positive correlations of MD with TMT-A (*rho *= 0.17, *P *= 0.01) and TMT-B (*rho *= 0.20, *P *= 0.005), as well as negative correlations of MD with VF (*rho *= −0.22, *P *= 0.001) and WLR (*rho *= −0.23, *P *< 0.001) in the post-SARS-CoV-2 group only. All other correlations were nonsignificant (*SI Appendix*, Table S4 and Fig. S6).

Additional regression analyses with age as the predictor of free water and MD showed significant positive associations of age with both imaging markers, as well as group × age interactions indicating stronger effects in post-SARS-CoV-2 individuals (please refer to *SI Appendix*, Table S5 and Fig. S7, for more detail).

### Prediction of a Past SARS-CoV-2 Infection Based on Imaging Markers.

**Fig. 5.**
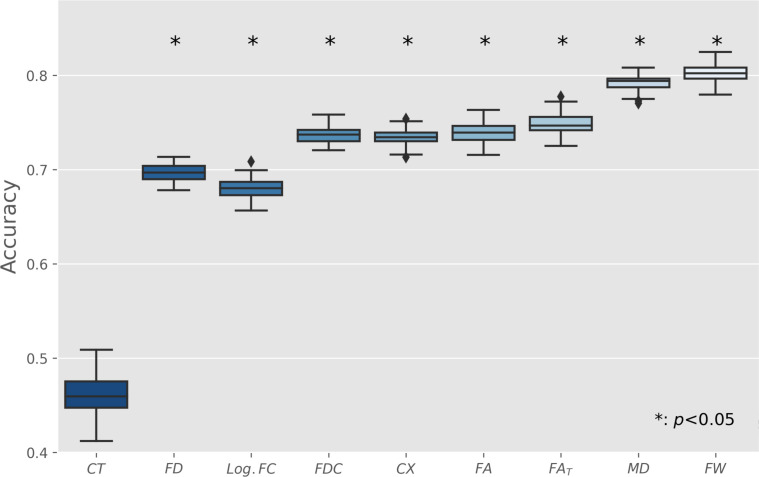
Prediction of past SARS-CoV-2 infection based on imaging markers. To assess the diagnostic relevance of evaluated brain imaging markers, a supervised machine learning analysis was performed. The displayed boxplots represent the accuracy of logistic regression models trained on regional imaging metrics to predict past SARS-CoV-2 infection. Model training occurred in a 10-fold nested cross-validation setup and was repeated 100 times for each marker with different random split regimens. Asterisks indicate significant difference to null model predictions, which were based on group label permutation. Predictive models based on free water and MD reached a considerable accuracy of approximately 80% in predicting a past SARS-CoV-2 infection. By that, they outperformed other imaging markers under study. All models except those based on cortical thickness achieved a significantly better performance than null models. *Abbreviations*: CT = cortical thickness, CX = complexity, FA = fractional anisotropy, FA_T_ = FA of the tissue, FD = fiber density, FDC = fiber density and cross-section, FW = free water, Log. FC = logarithm of fiber cross-section, MD = mean diffusivity, PSMD = peak width of skeletonized MD, WMH = white matter hyperintensity.

We examined the predictive capacity of derived imaging markers employing a supervised machine learning approach (Fig. 5). Free water (80.21%) and MD (79.38%) achieved the strongest median prediction accuracies. The median cortical thickness score was 45.95%. All investigated metrics but cortical thickness scored significantly better than null models for which the group assignment was randomly permuted.

### Sensitivity Analyses.

Analysis results remained stable if formerly hospitalized post-SARS-CoV-2 individuals were excluded and if post-SARS-CoV-2 individuals were stratified by recruitment route (*SI Appendix*).

## Discussion

We investigated brain structural alterations and neuropsychological sequelae in a large sample of individuals who recovered from mainly mild to moderate COVID-19. At median 289 d after the acute infection, these individuals showed significantly higher average free water and MD in the white matter compared to matched healthy controls. In contrast, cortical thickness and markers of cerebral small vessel disease were not significantly different between groups. In addition, white matter diffusion indices successfully predicted a past SARS-CoV-2 infection. We did not detect neuropsychological deficits in post-SARS-CoV-2 individuals. Collectively, our study suggests that a mild to moderate SARS-CoV-2 infection is associated with subtle microstructural alterations in the cerebral white matter beyond the stage of acute infection.

A key aspect of COVID-19 neuropathology appears to be the dynamic response of the intrathecal immune system to the virus. Evidence of neuroinflammation was reported in histopathological and clinical studies of COVID-19 patients: virus invasion (24, 25), activation of glial cells (9, 26, 27), and a cytokine response in the cerebrospinal fluid accompanying neurological and psychiatric COVID-19 symptoms (10). In our study, we observed widespread increases of extracellular free water and MD in post-SARS-CoV-2 individuals encompassing all brain lobes. Supplementary analyses showed that these increases relate to approximately 7 “years of healthy aging” indicating a biologically relevant effect. Both free water and MD are sensitive to an activated immune response causing excessive extracellular free water and thus increased diffusivity (28–30). More specifically, microglia and astrocytes emit cytokines upon activation, inducing osmosis of water from the blood into the extracellular space (31, 32). Interestingly, endothelial dysfunction and subsequent vascular leakage due to persistent immune activation have been previously implicated in the pathophysiology of COVID-19 (33, 34). Taken together, it is conceivable that the observed increase in free water and MD could be an indirect sign of a prolonged neuroinflammatory reaction to a SARS-CoV-2 infection. Nevertheless, other possible mechanisms for changes in the extracellular space need to be considered.

Volume increases in the extracellular compartment might be accompanied by structural damage like demyelination as well as axonal disruption secondary to neuroinflammation. Free-water corrected diffusion markers enable further guidance in microstructural interpretations. While analyses of overall mean values showed no significant group differences in free-water corrected FA_T_, voxel-wise statistics identified increased FA_T_ in corresponding frontal areas of post-SARS-CoV-2 individuals. Yet, these changes included only ~3% of the white matter skeleton, indicating either subtle or spatially limited effects localized to association tracts. Normal to increased FA_T_ in the presence of elevated free water suggests minor microstructural alterations like axonal compression or displacement rather than damage to myelin sheaths or axons which would rather lead to FA_T_ decreases (21). Moreover, fixel markers, which also model properties of the tissue compartment (35, 36), did not show group differences averaged across the entire white matter skeleton. Thus, in contrast to previous work demonstrating more widespread FA reductions in small samples of hospitalized COVID-19 cases (37–39), our findings suggest that white matter changes following a mild to moderate SARS-CoV-2 infection most likely reflect subtle increases in extracellular free water as opposed to structural neural damage.

Based on previous histopathological reports of vasculopathy in COVID-19 and higher ACE-2 expression in cells of the blood–brain barrier, we hypothesized that post-SARS-CoV-2 individuals would show alterations in imaging markers of small vessel disease burden (14). However, in our study, WMH (white matter hyperintensity) load was not significantly different, indicating that a mild to moderate course of COVID-19 does not lead to visually accessible vascular lesions (WMH) as previously reported (40). PSMD, an established imaging marker of small vessel disease more sensitive to microstructural changes (23), showed nominally higher values in post-SARS-CoV-2 individuals, but differences did not survive Bonferroni correction. Taken together, follow-up investigations are needed to understand whether subtle long-term vascular impairments will eventually increase the prevalence of cerebrovascular disease among COVID-19 convalescents (33).

Alterations of cortical gray and white matter commonly co-occur in neurological diseases (41, 42). Notably, this was not the case in our study. This is contrasted by a recent report on mildly affected COVID-19 subjects in the UK Biobank demonstrating longitudinal volumetric reductions in the gray matter in olfactory networks (11). On the other hand, a current cross-sectional study has shown gray matter volume increases in long-COVID patients compared to healthy controls (43). The discrepancies between these studies and our work may be due to general differences in recruitment strategies (general population vs enriched samples of individuals suffering from long-term sequelae) and study designs (longitudinal vs. cross-sectional), both of which likely affect the sensitivity to detect gray matter changes associated with a SARS-CoV-2 infection.

By providing scores of prediction accuracy, our machine learning analysis evaluated brain imaging markers for their diagnostic relevance. Logistic regression models based on free water and MD achieved a considerable accuracy of ~80% in predicting a past SARS-CoV-2 infection, outperforming other imaging markers under study. Of note, cortical thickness achieved the lowest accuracy, not significantly differing from prediction by chance. The difference in accuracy between diffusion metrics and cortical thickness might highlight that in mild to moderate COVID-19, pathophysiological aspects are better detected by diffusion imaging–based techniques. Finally, the higher accuracy of fiber cross-section compared to cortical thickness—both morphometric measures—might imply that COVID-19-associated alterations preferably occur in the white matter.

We want to emphasize that our results represent average effects, i.e., not every mild to moderate affected COVID-19 patient may exhibit the reported changes. In addition, our results are based on a nonvaccinated cohort. As vaccination has been repetitively demonstrated to be a highly effective measure against COVID-19, vaccinated patients possibly exhibit less of the pathophysiological substrates identified in our study (44).

It is important to put the observed brain white matter alterations into a clinical perspective. Reported persisting clinical sequelae of COVID-19 include executive dysfunction, anxiety, depression, fatigue, muscle weakness, and sleep impairment (11, 45–47). In contrast, we found no significant difference for any cognitive domain, depression, anxiety, and neurological symptoms between groups. Similar to our previous finding in a larger, yet overlapping sample (1), nominally, post-SARS-CoV-2 individuals showed even better performances in MMSE, CDT, and VF compared to matched controls. Besides the absence or mild expression of the respective symptoms, other reasons, such as our relatively long follow-up period and a potential selection bias of highly motivated post-SARS-CoV-2 participants, as well as differing degrees of social deprivation as a result of country-specific pandemic control measures, may explain the discrepancy with other studies. Exploration of imaging–behavior interactions showed that relatively increased free water and MD were associated with worse executive performance (TMT-A/B), working memory (WLR), and VF in post-SARS-CoV-2 individuals, thus providing a preliminary pathophysiological link between neurocognitive deficits and brain alterations in individuals recovered from COVID-19. Clearly, more research is needed to increase our understanding of factors underlying the persistence of neurological symptoms in a subgroup of COVID-19 patients.

Strengths of this work lie in its considerable sample size; high-quality imaging and phenotypical data; a robust and reproducible image processing pipeline; the investigation happening at an early stage of the pandemic, potentially alleviating the problem of different COVID-19 strains and vaccinations as confounders; and a conservative statistical correction scheme to reduce the false-positive rate.

However, our study also exhibits limitations. Our investigation lacks information about SARS-CoV-2 strains as well as precise disease severity stratification beyond the self-reported information on hospitalization and subjective perception of disease intensity. In addition, we follow a cross-sectional observational study design unable to fully address premorbid group differences despite a rigorous matching procedure and insufficient to infer causality. Future longitudinal studies could not only elaborate on the trajectory of the identified microstructural white matter alterations but also address the question whether these findings are markers of increased susceptibility for the development of neurological sequelae. Finally, our correction scheme for multiple testing may not only be regarded as a study strength but also as overly conservative, considering the potential statistical dependencies between variables. We opted for this strategy, as we prioritized the minimization of false-positive findings. Nevertheless, we did report uncorrected *P* values and recognized nominal group differences in clinical and imaging markers to encourage future hypothesis-driven studies on neurological manifestations of COVID-19 with less conservative approaches.

We performed a comprehensive assessment of established neuroimaging markers for structural neural integrity to characterize neurobiological changes potentially underlying postacute COVID-19 neuropsychological sequelae after a mainly mild to moderate disease course. Our findings support the notion of a prolonged neuroinflammatory response indicated by subtle but widespread increases in extracellular free water and mean diffusivity in the white matter of COVID-19 convalescents. In contrast, we did not observe signs of cortical atrophy or macrostructural vascular damage. Importantly, despite identifying this characteristic imaging footprint, the investigated sample exhibited no marked neuropsychological symptoms 10 mo after SARS-CoV-2 infection. External validation and longitudinal investigations are needed to further clarify the clinical relevance of our findings.

## Materials and Methods

### Study Population.

We examined participants of the HCHS COVID Program. A detailed description of the study design has been published previously (1). Post-SARS-CoV-2 participants 1] had a positive PCR test for SARS-CoV-2 and 2] were aged between 45 and 74 y at inclusion. Recruitment routes included both invitation upon laboratory-confirmed SARS-CoV-2 infection and self-referral of participants following newspaper announcement. Subsequent to recruitment, the participants underwent the study protocol of the HCHS (48)—including cranial MR imaging, neuropsychological testing, and a self-report questionnaire on COVID-19-associated symptoms. In addition, a healthy control group was sampled from the original HCHS cohort which was assessed prior to the SARS-CoV-2 pandemic (48). The previously reported matching procedure (1) considered confounders allowing for a comprehensive investigation of COVID-19 pathophysiology in multiple organ systems beyond the brain, including the lungs, heart, vasculature, and kidneys. In contrast to the previous procedure, we performed a 1:1 propensity score matching specifically accounting for confounds known to affect cognitive performance as well as indices derived from structural and diffusion MR imaging: the groups were matched for age, sex, and years of education as well as for the prevalence of arterial hypertension, diabetes mellitus, dyslipidemia, and smoking behavior using the matchit (v4.3.3) R package (49).

### Ethics Approval.

The local ethics committee of the Landesärztekammer Hamburg (State of Hamburg Chamber of Medical Practitioners, PV5131) approved the study, and written informed consent was obtained from all participants (1, 50).

### Clinical Assessments.

Cognitive testing was performed by a trained study nurse and included the MMSE (51), TMT-A/B (52), VF, and WLR subtests of the Consortium to Establish a Registry for Alzheimer’s Disease Neuropsychological Assessment Battery (CERAD-Plus) (53), as well as the CDT (54). Psychosocial symptom burden was evaluated by the GAD-7 (anxiety) (55) and PHQ-9 (depression) (56). Moreover, self-reported neurological symptoms (headache, dizziness, fatigue, and sleep disturbances) were assessed by part of the PHQ-15 (57).

### Brain Imaging.

Image acquisitions have been described in detail before (50). Put briefly, 3D T1-weighted rapid acquisition gradient-echo sequence (MPRAGE, 0.83 × 0.83 × 0.94 mm), 3D T2-weighted FLAIR (0.75 × 0.75 × 0.9 mm), and single-shell diffusion MRI (2 × 2 × 2 mm, 64 noncollinear gradient directions, b = 1,000 s/mm^2^) were acquired on a single 3T Siemens Skyra MRI scanner (Siemens, Erlangen, Germany). Detailed parameters can be found in *SI Appendix*.

An overview of the derived imaging markers for the gray and white matter can be found in Fig. 1. For a detailed account on image preprocessing, derivation of morphometric and diffusion indices, and QA, please refer to *SI Appendix* (58).

Following preprocessing, we derived conventional DTI markers of white matter microstructure, i.e., FA and MD, which have been extensively used in neuroscientific and neuropsychological research (59, 60). Free-water imaging was employed to model an extracellular free-water compartment, sensitive to immune activation (61) and atrophy (62), as well as a cellular tissue compartment (FA_T_), more closely reflecting myelin and axonal alterations than their DTI equivalents (28). Fixel-based analysis, a multitissue model addressing more complex white matter compositions, was used to derive metrics of fiber density, fiber-bundle cross-section (FC), fiber density and cross-section (FDC), and complexity (63). For further statistical analysis, diffusion markers were averaged across a representative skeleton of the entire white matter derived by tract-based spatial statistics as well as within 71 anatomical white matter fiber tracts reconstructed with TractSeg (64, 65). Finally, PSMD, a surrogate marker of cerebral small vessel disease, was calculated (23).

After cortical surface reconstruction with the Computational Anatomy Toolbox for SPM (CAT12), mean cortical thickness was estimated as a proxy for neurodegenerative processes (20, 66, 67). Normalized volumes of white matter hyperintensities (WMH load) were obtained by FSL’s Brain Intensity AbNormality Classification Algorithm (BIANCA) with LOCally Adaptive Threshold Estimation (LOCATE) (68, 69).

### Statistical Analysis.

All statistical analyses were conducted in Python 3.9.1 (70, 71), CAT12 (66, 67, 72), as well as mrclusterstats (73). Statistical tests were two sided, with a *P *< 0.05 as significance threshold. In the case of averaged imaging and clinical data, *P* values were adjusted by Bonferroni correction for 11 and 9 comparisons, respectively. We chose this conservative correction scheme in order to minimize the possibility of false-positive findings. Additionally, sensitivity analyses were performed by 1] excluding post-SARS-CoV-2 individuals who had been hospitalized, and 2] stratifying the post-SARS-CoV-2 group by recruitment strategy, following the same procedures as described above.

#### Phenotypical data.

Sample characteristics were compared between healthy controls and post-SARS-CoV-2 participants using Χ^2^ tests (binary) and two-sample *t* tests (continuous). Clinical variables were compared between groups in separate analyses of covariance (ANCOVA) adjusted for age, sex, and education.

#### Imaging.

Statistical analysis of imaging parameters was conducted in two steps. First, global measures, i.e., mean skeletonized diffusion parameters, mean cortical thickness, WMH load, and PSMD, were compared between post-SARS-CoV-2 individuals and healthy controls in separate ANCOVAs, adjusted for age, sex, and education. In the case of FC and FDC, total intracranial volume served as an additional covariate. Next, in an effort to interrogate spatial patterns of brain structural alterations associated with a mild to moderate SARS-CoV-2 infection, we performed whole-brain voxel-wise permutation-based testing for skeletonized diffusion markers. Utilizing the same design matrices as in the ANCOVAs, we employed 5,000 permutations, threshold-free cluster enhancement, and family-wise error correction across multiple hypotheses. We supplemented voxel-wise statistics with a tract of interest approach by performing abovementioned ANCOVAs on the level of TractSeg-derived anatomical white matter tracts. Here, *P* values were adjusted by Bonferroni correction for 71 comparisons. Vertex-wise cortical thickness was statistically analyzed in a general linear model as implemented in CAT12 with family-wise error correction and a cluster threshold of 10.

#### Associations between clinical and imaging data.

In case of significant group differences of averaged imaging markers, we performed exploratory regression analyses testing for associations between these imaging markers and neuropsychological scores in the entire sample. Moreover, group × imaging marker interactions were included in the regression model, and post hoc Spearman correlations were conducted for each group separately. As imaging markers are known to change as a function of age, we performed additional regression analyses with age in the matched control group to derive beta estimates for conversion of group differences in units of “years of healthy aging” in order to aid biological interpretation of our results. As we deemed these analyses exploratory, no correction for multiple comparisons was performed.

### Machine Learning Prediction.

To further evaluate their predictive capacities, all brain imaging markers calculated in the study were averaged within regions of interest where applicable (Desikan–Killiany cortical atlas parcels and TractSeg-derived anatomical white matter tracts) and propagated to a comparative supervised machine learning pipeline (scikit-learn v1.0.2) (65, 74, 75). Per marker, multivariate logistic regression models were trained to predict past COVID-19. Models were scored with prediction accuracy, and statistical significance was assessed via comparison to null model predictions. Further details are provided in *SI Appendix*.

## Supplementary Material

Appendix 01 (PDF)

## Data Availability

Analysis codes and processed global imaging parameters data have been deposited in GitHub (See *SI Appendix*, Table S12; https://github.com/csi-hamburg/2022_petersen_naegele_postcovid_imaging/blob/main/global_imaging_markers.csv) (76). Personalized data from individual participants of the HCHS are not publicly available due to data protection regulations, but anonymized data can be accessed by interested researchers via a request to the HCHS steering committee based on a material transfer agreement.
